# PD-1 and CTLA-4 exert additive control of effector regulatory T cells at homeostasis

**DOI:** 10.3389/fimmu.2023.997376

**Published:** 2023-03-07

**Authors:** Joseph A. Pereira, Zachary Lanzar, Joseph T. Clark, Andrew P. Hart, Bonnie B. Douglas, Lindsey Shallberg, Keenan O’Dea, David A. Christian, Christopher A. Hunter

**Affiliations:** ^1^ Department of Pathobiology, University of Pennsylvania, Philadelphia, PA, United States; ^2^ Department of Immunology, Blavatnik Institute, Harvard Medical School, Boston, MA, United States; ^3^ Department of Medical Oncology, Dana-Farber Cancer Institute, Boston, MA, United States; ^4^ Department of Medicine, University of Pennsylvania, Philadelphia, PA, United States

**Keywords:** treg - regulatory T cell, checkpoint blockade immunotherapy, PD-1 - PD-L1 axis, CTLA-4 (cytotoxic T lymphocyte-associated antigen 4), homeostatic regulation, immune suppression, IL-10 (Interleukin 10), eTreg cells

## Abstract

At homeostasis, a substantial proportion of Foxp3^+^ T regulatory cells (T_regs_) have an activated phenotype associated with enhanced TCR signals and these effector T_reg_ cells (eT_regs_) co-express elevated levels of PD-1 and CTLA-4. Short term *in vivo* blockade of the PD-1 or CTLA-4 pathways results in increased eT_reg_ populations, while combination blockade of both pathways had an additive effect. Mechanistically, combination blockade resulted in a reduction of suppressive phospho-SHP2 Y580 in eT_reg_ cells which was associated with increased proliferation, enhanced production of IL-10, and reduced dendritic cell and macrophage expression of CD80 and MHC-II. Thus, at homeostasis, PD-1 and CTLA-4 function additively to regulate eT_reg_ function and the ability to target these pathways in T_reg_ cells may be useful to modulate inflammation.

## Introduction

At homeostasis, Foxp3^+^ regulatory T cells (T_reg_) (1), have a critical role in prevention of auto-immunity and can limit the intensity and duration of inflammatory responses (2–4), T_reg_ cells can originate from the thymus (nT_reg_), or naïve CD4^+^ T cells that receive TCR stimulation combined with signals from transforming growth factor beta (TGFβ) and IL-2 can lead to Foxp3 expression and the formation of induced T_reg_ cells (iT_reg_) (5, 6). T_reg_ cells differ from conventional CD4^+^ and CD8^+^ T cells (T_conv_), in that the majority of them have a TCR that recognizes self-antigens and are specialized to preserve tolerance (7–9). It is now appreciated that T_reg_ cells require ongoing TCR activation and costimulation to retain Foxp3 expression, suppressive capacity (10), and survival (11–13). This is illustrated by the spontaneous immunopathology in experimental models when T_reg_ cells are absent (14–17). The clinical relevance of T_reg_ cell mediated control of adaptive responses is illustrated by X-linked immunodysregulation polyendocrinopathy and enteropathy (IPEX). In these patients, the Foxp3 gene is mutated and has an impaired ability to drive T_reg_ formation, resulting in autoimmune diseases such as neonatal type 1 diabetes, hemolytic anemia, eosinophilia, and hyper IgE production (18).

Given the role of T_reg_ cells in limiting immune responses there is considerable interest in promoting their activities to limit inflammation while the ability to antagonize T_reg_ cells is one approach to augment anti-tumor responses (19–21). There is considerable heterogeneity in T_reg_ cell populations associated with development (iT_reg_ versus nT_reg_), activation status and their responses to inflammation (22, 23). This is illustrated by the description of central T_reg_ (cT_reg_) and effector T_reg_ cells (11, 22) (eT_reg_) as distinct populations defined based on activation status (11). There is emerging evidence that the relative ratio of effector T_conv_ cells: T_reg_ cells is an important determinant for the outcome of immunotherapy in cancer (24). However, too many T_reg_ cells can be deleterious and lead to reduced effector responses in the context of infection or cancer (21, 25). Consequently, there need to be processes to balance T_reg_ cell activities and IL-2 availability is one mechanism involved in modulation of the T_reg_ cell pool (26, 27). There is also evidence that the inhibitory receptors PD-1 (28, 29) and CTLA-4 (20) restrict T_reg_ cell activities in the setting of cancer, autoimmunity and infection (20, 21, 29).

PD-1 and CTLA-4 are expressed by activated T cells and most studies on these pathways have focused on their impact on effector responses which has formed the basis for checkpoint blockade in cancer. In this context, there is evidence that PD-1 and CTLA-4 act in cis and engage SHP2 phosphatases (30–33) which antagonize TCR signals (34–36), and thus blunt the response of effector T cells (37). In addition, the ability of the extracellular domain of CTLA-4 to sequester CD80/86 provides an additional trans mechanism to limit professional antigen presenting cell (APC) function required for optimal effector T cell activities (34). A subset of T_reg_ cells also express these receptors (21, 29), and several reports have highlighted that effector T_reg_(eT_reg_) cells express the highest levels of PD-1 and CTLA-4 (28, 29). It appears that while eT_reg_ cells receive continuous TCR signals, constitutive signals through PD-1 constrain the size of the eT_reg_ cell pool (28, 29). In contrast to PD-1, T_reg_ cell expression of CTLA-4 provides an effector mechanism that can limit autoimmune inflammation, but total loss of CTLA-4 results in enhanced T_reg_ cell populations (38, 39) and lineage specific deletion of CTLA-4 in T_reg_ cells results in enhanced T_reg_ cell activities in models of autoimmunity (20). Interestingly, while CTLA-4 is a relevant target to enhance effector responses during cancer in some tumor models (33, 40), blockade of CTLA-4 results in enhanced costimulatory signals and hyperproliferation of T_reg_ cells which drove increased immune tolerance (41).

Since a subpopulation of T_reg_ cells co-express PD-1 and CTLA-4 (21, 29), the finding that even short-term blockade of PD-L1 result in increased eT_reg_ cell population at homeostasis raises questions about the relationship between the PD-1 and CTLA-4 pathways. For example, it is unclear if these pathways are both constitutively active, act together or separately or are functionally redundant at stasis and whether mitigation of these checkpoint proteins would impact the ratio of cT_reg_: eT_reg_ cell populations. The studies presented here reveal at homeostasis that the combined blockade of PD-1 and CTLA-4 have an additive effect on expansion of eT_reg_ cell populations associated with reduced APC function. Thus, PD-1 and CTLA-4 have distinct but complementary roles in the tonic regulation of T_reg_ cell homeostasis.

## Materials and methods

### Mice

All mice used were housed in the University of Pennsylvania Department of Pathobiology vivarium with 12 hour light and dark cycles, maintained at temperature ranges of 68°F - 77°F and humidity ranges from 35% - 55% humidity in accordance with institutional guidelines. C57BL/6 mice were purchased from Taconic (Rensselaer, NY, USA) at 6 weeks of age and housed in the University of Pennsylvania Department of Pathobiology vivarium for 2 – 4 weeks until used.

Ethical oversight of all animal use in this study was approved by the University of Pennsylvania Institutional Animal Care and Use Committee.

### Homeostatic *in vivo* combination checkpoint blockade

*In vivo blockade antibodies:* Details of antibodies and reagents in blockade can be found in **Supplementary Table 1**.

Inhibition of PD-1/PD-L1 signaling was performed by intraperitoneal injection of 1mg/dose of αPD-L1 (clone: 10F.9G2, BioXcell) supplemented with 500μg/dose of polyclonal hamster IgG isotype (clone: polyclonal Armenian hamster, BioXcell). Inhibition of CTLA-4 signaling was performed by intraperitoneal injection of 500μg/dose of αCTLA-4 (clone: UC10-4F10-11, BioXcell) supplemented with 1mg/dose of IgG2b isotype (clone: LTF-2, BioXcell) while control mice were treated with 1mg/dose IgG2b isotype supplemented with 500μg/dose of polyclonal hamster IgG isotype. Mice were sacrificed 72 hours following treatment and splenocytes were analyzed *via* flow cytometry.

### Vaccine-induced immune responses during checkpoint blockade

8-week-old C57BL/6 mice were treated with either αPD-L1, αCTLA-4, combination αPD-L1 and αCTLA-4, or combination isotype antibody mixes (same dosages/combinations/antibody clones used in the homeostatic blockade above). After 72 hours, congenically labeled CD45.1^+^ OTI cells were isolated from healthy donor spleen using an Easysep Mouse CD8^+^ T cell isolation kit (19853, STEMCELL Technologies). 5,000 OTI cells were injected intraperitoneally into the antibody-blockade treated hosts. After 24 hours following the transfer of OTI cells, we intraperitoneally vaccinated these mice with 200,000 tachyzoites of a non-replicating vaccination-strain of *T. gondii* that expresses OVA (CPS-OVA). Previous studies have shown that CPS alone does not lead to activation of OTI or P14 TCR transgenic CD8^+^ T cells, and expression of OVA is essential for activation and expansion of the OTI T cells (42). At 24 hours after vaccination, we re-dosed these groups of mice with the original blocking antibody they had previously received to maintain the blockade treatment. At 7 days post-vaccination, the spleen, peritoneal exudate cells (PEC), and draining lymph nodes (mediastinal LN) were analyzed *via* flow-cytometry to assess the impact of blockade on the formation of an OTI response, endogenous responses to the parasite itself, and the phenotypes of the Tregs in these tissues.

### Tacrolimus treatment

FK506 (F4679-5MG, Sigma-Aldrich, MO, USA) was reconstituted in DMSO to 25mg/ml, and then the reconstituted stock was diluted in 1xDPBS to achieve a working concentration of 2.5mg/ml. 8-week-old C57BL/6 mice were subcutaneously injected with 50µl of FK506 at 2.5mg/ml to deliver 125µg of FK506 per dose daily of either FK506 or PBS vehicle control every 24 hours over a 96 hour period. Following 96 hours of treatment, splenocytes were then harvested and analyzed *via* flow cytometry.

### Isolation of tissues for analysis

*Tissue Preparation:* Single cell suspensions were prepared from spleen for flow cytometry analysis. Spleens were mechanically processed and passed through a 70µm nylon filter and then lysed in 1ml of 0.846% solution of NH_4_Cl for red blood cell lysis. The cells were then washed in cRPMI and stored on ice.

### Analysis by flow cytometry

*Staining antibodies and staining reagents:* Antibody, viability dye, Fc block, dilutions, and buffer reagent details can be found on **Supplemental Table 1**.

*T cell staining:* Aliquots consisting of 5e6 cells were washed with ice cold 1xDPBS in a 96 well round bottom plate, then incubated in in 50µl volume of viability stain reconstituted in 1xDPBS for 20 minutes on ice and then washed in 0.2% FACS buffer. The cells were then incubated in 50µl volume of Fc block for 30 minutes on ice. In the event of vaccination, the cells were washed in 0.2% FACS buffer and then stained with in 50µl volume of 0.2% FACS buffer supplemented with tetramer loaded with the parasite-specific peptide AS15 (43) for 30 minutes on ice, in non-vaccination studies this step was skipped. The cells were washed in 0.2% FACS buffer, and then incubated for 30 minutes on ice in 50µl volume of antibody cocktail composed of surface-stain antibodies in 0.2% FACS buffer supplemented with brilliant stain buffer (**Supplemental Table 1**). The cells were washed in 0.2% FACS buffer and re-suspended in 100µl Foxp3 Perm-fix cocktail (00-5523-00, Thermo Fisher Scientific) for 4 hours at 4°C. The cells were then washed twice in 1X permeabilization buffer, and then re-suspended in an intracellular staining cocktail composed of intracellular-stain antibodies diluted in 1x permeabilization buffer supplemented with normal goat serum of for 2 hours at 4°C. The cells were then washed with 1x permeabilization buffer twice, and then resuspended in 50µl of Goat α-Rabbit detection antibody diluted in 1X permeabilization buffer for 2 hours at 4°C. The cells were washed in 1x permeabilization buffer and resuspended in 500µl 0.2% FACS buffer for flow cytometric analysis.

*Cytokine staining:* To detect intracellular cytokines on T cells, cells were re-suspended in a 1X dilution of Cell Stimulation Cocktail Plus Protein Transport Inhibitors (Invitrogen, #00-4975-93, CA) in cRPMI for 2 hours at 37°C and 5% CO_2_. Cells were then washed, surface stained, and permeabilized as described above in the T cell panel. The cytokine stain prepped cells were then intracellularly stained with a cytokine detection panel for 2 hours on ice. The cells were washed and then resuspended in 500µl 0.2% FACS buffer for analysis.

*Myeloid staining:*Aliquots of 5e6 cells were washed in ice cold 0.2% FACS buffer in a 96 well and then viability stained and Fc-blocked as described in the T cell panel. The cells were surface stained in 50µl of antibody cocktail consisting diluted in 0.2% FACS buffer supplemented with brilliant stain buffer on ice for 30 minutes. The cells were washed and fixed in with 2% PFA (15710-S, Electron Microscopy Sciences) diluted in 0.2% FACS buffer for 15 minutes at room temperature. The cells were then washed and then re-suspended in 500µl 0.2% FACS buffer for analysis.

*Phos-flow:* Splenocyte-derived CD4^+^ T cells were isolated using Easysep Mouse CD4^+^ T cell isolation kit (19852, STEMCELL Technologies), and then 2e5 cells/well were plated in a 96 well plate, and viability stained as described above using sterile 1xDPBS. Cells were blocked for PD-1, CTLA-4, or combination of PD-1 and CTLA-4 using anti-PD-1 (clone: RMP1-14, BioXcell), anti-CTLA-4 (clone: UC10-4F10-11, BioXcell) or isotype control antibodies (clone: 2A3, BioXcell, and clone: polyclonal Armenian hamster IgG, BioXcell). The cells were blocked in 100µl of PD-1/CTLA-4 blocking cocktails in sterile MACS buffer (2% FCS, 2mM EDTA, in 1xDPBS) at a concentration of 10µg/ml of antibody on ice for 20 minutes. The cells were washed with sterile MACS and were then resuspended in 100µl sterile RPMI containing 0.5% BSA, and then transferred to a 96 well plate that had been coated overnight at 4°C with 5µg/ml αCD3 (BE0001-1, BioXcell), 5µg/ml CD80-Fc (555404, Biolegend), and 2µg/ml PD-L1-Fc (758206, Biolegend). The cells were either incubated at 37°C for 30 minutes or one hour, and then mixed with 100µl of 5% PFA (15710-S, Electron Microscopy Sciences) diluted in ice cold 1xDPBS and incubated on ice for 20 minutes (direct exvivo phos-flow assessments were directly fixed without incubation). The cells were washed 2x in 1xDPBS, and permeabilized in 100µl Foxp3 Perm-fix cocktail (00-5523-00, Thermo Fisher Scientific) for 2 hours, and then washed as described above. The cells were re-suspended in an intracellular staining cocktail composed of intracellular-stain antibodies diluted in 1x permeabilization buffer for 2 hours at 4°C. The cells were washed twice in 1x permeabilization buffer and resuspended in 0.2% FACS buffer for flow cytometric analysis.

*Data acquisition:* The cells were analyzed on a FACS Symphony A5 (BD Biosciences) using BD FACSDiva v9.0 (BD Biosciences) and analysis was performed with FlowJo (10.8.1, BD biosciences).

*Statistics:* Statistical analysis was performed using Prism 9 for Windows (version 9.2.0). For comparison of means between two groups, either a two-tailed unpaired, or paired student’s *t* test was utilized with a 95% CI depending on separate treatment groups or treatments within groups. Analysis for univariate statistics comparing multiple means was performed using a one-way ANOVA (family-wise significance and confidence level of 95% CI), with *post-hoc* analysis consisting of Fisher’s LSD test for direct comparison of two means within the ANOVA, or Tukey’s multiple comparisons test for comparisons of all means within the test group for multiple-comparison correction. For multi-group multivariate analysis, a two-way ANOVA with *post-hoc* analysis utilizing Sidak’s multiple comparisons test for comparisons across two groups with two variables, or Tukey’s multiple comparisons test for comparisons across multiple groups for multiple variables (also with a 95% CI). Probability for *p* values <0.05 or lower were considered statistically significant. All error bars in the figures indicate standard error of the mean (SEM).

*UMAP analysis:* Uniform Manifold Approximation and Projection for Dimension Reduction (UMAP) analysis was performed using the UMAP plug-in using the Euclidean distance function with a nearest neighbor score of 20, and a minimum distance rating of 0.5 (version: 1802.03426, 2018, ^©^2017, Leland McInness) for Flowjo (Version 10.8.1). All stained parameters were included in UMAP analysis except for: Live Dead (gated out), CD4 (pre-gated), PD-L1 and CTLA-4 (avoiding grouping bias), Foxp3 (avoiding grouping bias or already pre-gated). The heatmap overlay figures for UMAP analysis presented are based on median fluorescence of each labeled stain in each figure and generated within Flowjo (Version 10.8.1).

*Data availability statement:* The data that support the findings of this study are available on request from the corresponding author C.A. Hunter.

## Results

### Preferential expression of PD-1 and CTLA-4 by eT_reg_ cells

**Figure 1:**
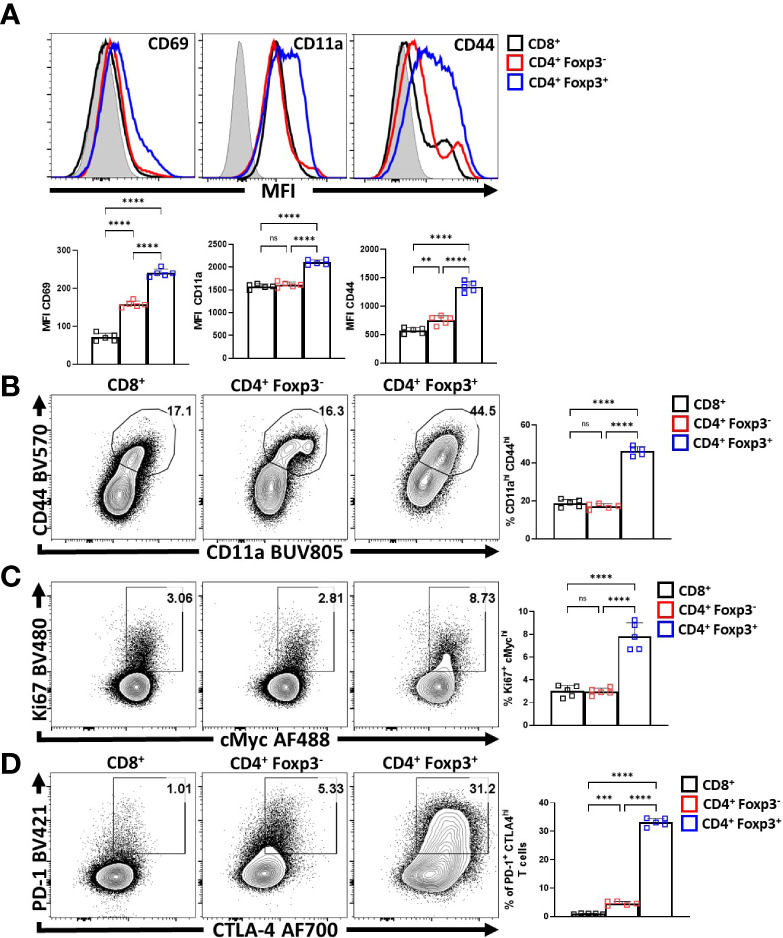
T_reg_ cells are the most active and proliferative T cells at homeostasis, yet express PD-1 and CTLA-4. Splenocytes from naïve 8-week old male C57BL/6 mice were analyzed via high-parameter flow cytometry to compare the expression of activation, proliferation, and PD-1/CTLA-4 proteins CD8^+^, CD4^+^ Foxp3^-^ (CD4 T_conv_), and CD4^+^ Foxp3^+^ (T_reg_) cells for the following figures. **(A)** Histogram comparisons of gMFI of CD69, CD11a, and CD44 expression between the CD8/CD4^+^ T_conv_ and T_reg_ compartments (n = 5/group, 1-way ANOVA with Tukey’s multiple comparisons test, **p < 0.01, ****p < 0.0001, 4 experimental replicates). **(B)** Flow plots of *ex-vivo* CD11a and CD44 staining comparing the proportion of CD11a^hi^ CD44^hi^ cells within each subset (n = 5/group, 1-way ANOVA with Tukey’s multiple comparisons test, ****p < 0.001, 4 experimental replicates). **(C)** Plots of depicting comparisons of the proportion of Ki67^+^ cMyc^hi^ cells across these subsets (n = 5/group, 1-way ANOVA with Tukey’s multiple comparisons test, ****p < 0.0001, 4 experimental replicates). **(D)** Plots demonstrating proportions of PD-1^+^ and CTLA-4^hi^ cells between the T_conv_ and T_reg_ compartments (n = 5/group, 1-way ANOVA with Tukey’s multiple comparisons test, ***p < 0.001, ****p < 0.0001, 4 experimental replicates). All data presented are means +/- SD and show individual data points. ns, not significant.

**Figure 2:**
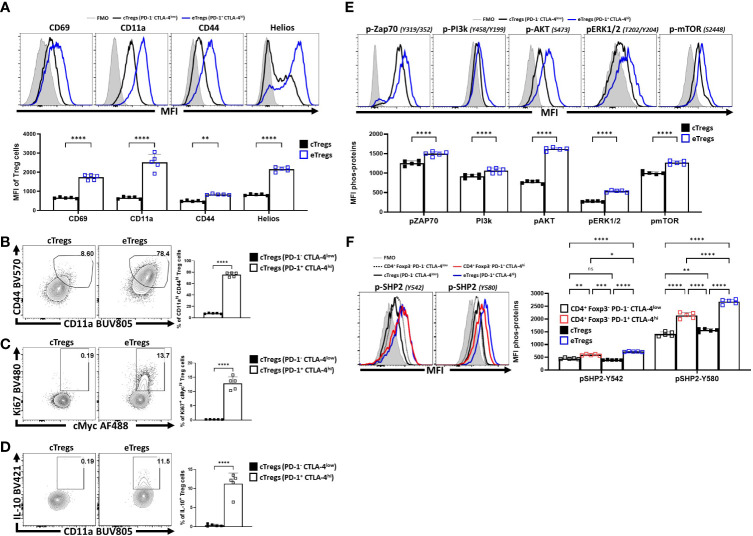
PD-1^+^ CTLA-4^+^ eT_reg_ cells express Helios and have activated T_reg_ effector phenotypes compared to PD-1^-^ CTLA-4^-^ cT_reg_ cells. Splenocytes from naïve 8-week old male C57BL/6 mice were analyzed via high-parameter flow cytometry and then pre-gated (**Supplemental Figure 1B**) on PD-1^+^ CTLA-4^hi^ (eT_reg_) vs PD-1^-^ CTLA-4^low^ (cT_reg_) subsets. Phenotypes were compared between the c/eT_reg_ subsets based on the expression of proteins associated with activation, proliferation, and IL-10 production. Additionally, TCR-downstream phosphorylation potential in response to activation between T_reg_ subsets was also evaluated. **(A)** Comparative histograms of CD69, CD11a, CD44, and Helios between eT_reg_ and cT_reg_ subsets demonstrating greater expression of activation associated proteins and Helios on eT_reg_ cells (n = 5/group, 2-way ANOVA with Sidak’s multiple comparisons test, **p < 0.01, ****p < 0.0001, 4 experimental replicates). **(B)** *Ex-vivo* flow-plots comparing the proportion of CD44^hi^ CD11a^hi^ populations and Ki67^+^ cMyc^hi^ populations **(C)**, between cT_reg_ and eT_reg_ subsets (n = 5/group, two-tailed unpaired student’s t-test, ****p < 0.0001 4 experimental replicates). **(D)** Flow plots following PMA/Ionomycin stim comparing the proportion of IL-10^+^ CD11a^hi^ cells between cT_reg_ and eT_reg_ subsets (n = 5/group, two-tailed unpaired student’s t-test, ****p < 0.0001 4 experimental replicates). **(E)** Histogram comparisons of gMFI of p-ZAP70, p-AKT, pERK1/2, and p-mTOR of cT_reg_ and eT_reg_ cells exvivo, demonstrating a greater magnitude of phospho-protein presence in eT_reg_ cells comparatively (n = 5/group, 2-way ANOVA with Sidak’s multiple comparisons test, ****p < 0.0001, 2 experimental replicates). **(F)** Histogram comparisons of gMFI of p-SHP2 for Y580 and Y542 residues *exvivo* on PD-1^+^ CTLA-4^hi^ and PD-1^-^ CTLA-4^low^ CD4^+^ T_conv_ subsets, in addition to cT_reg_, and eT_reg_ cells (n = 5/group, 2-way ANOVA with Tukey’s multiple comparisons test, *p < 0.05, **p < 0.01, ***p < 0.001, ****p < 0.0001, 2 experimental replicates). All data presented are means +/- SD and show individual data points.

To compare the relative activation state of CD8^+^ T cells, CD4^+^ Foxp3^-^ T cells (T_conv_) and CD4^+^ Foxp3^+^ cells (T_reg_s) at homeostasis, the levels of CD69, CD11a, and CD44 (markers associated with TCR activation) were assessed. T_reg_ cells had highest expression of CD69, CD11a, and CD44 (**Figure 1A**; **Supplemental Figure 1A**), and the highest proportion of CD11a^hi^ CD44^hi^ cells (**Figure 1B**). Likewise, T_reg_ cells also had the largest proportion of Ki67^+^ and cMyc^+^ cells, two markers associated with proliferation (44, 45) (**Figure 1C**). These markers of activation and proliferation correlated with the preferential co-expression of PD-1 and CTLA-4 by T_reg_ cells compared to non-T_reg_ T cells (**Figure 1D**). Next, T_reg_ cells were divided into PD-1^-^ CTLA-4^low^ and PD-1^+^ CTLA-4^hi^ T_reg_ cells (**Supplemental Figure 1B**), that correlate with cT_reg_ and eT_reg_ subsets (11, 29) respectively. Based on this division, eT_reg_ cells had significantly greater expression of CD69, CD11a, CD44, and Helios (**Figure 2A**) and the eT_reg_ subset was enriched for cells that co-expressed elevated levels of CD11a and CD44 (**Figure 2B**), Ki67 and cMyc (**Figure 2C**). In addition, this eT_reg_ subset had an increased ability to produce IL-10 (**Figure 2D**). We also noted that the proportion of these proliferative eT_reg_ cells increased with age and could be as high as 40% of the T_reg_ cells in older mice (**Supplemental Figure 1C**).

Next, eT_reg_ and cT_reg_ cells, directly isolated from spleens, without any additional TCR activation, were stained for phosphorylation of TCR-associated proteins (ZAP70, PI3k, AKT, ERK1/2, and mTOR) and the SHP2 tyrosine sites Y542 and Y580, [of which Y542 can dephosphorylate Y580 - the active tyrosine site associated with inhibition of TCR signals (32, 46)]. As expected, in this setting, cT_reg_ cells had minimal signs of TCR activity when compared to eT_reg_ cells (**Figure 2E**). Regarding SHP2-Y542 and SHP2-Y580, comparisons of phospho-protein were made between PD-1^+^ CTLA-4^hi^ and PD-1^-^ CTLA-4^low^ subsets for both the CD4^+^ T_conv_ (Foxp3^-^) and T_reg_ (Foxp3^+^) T cell populations (**Figure 2F**). For this analysis, the lowest levels of pY542 and pY580 were detected in cT_reg_ and naïve T_conv_ cells whereas eT_reg_ cells had the highest levels of pY542 and pY580 SHP2. This was apparent even when comparing effector PD-1^+^ CTLA-4^hi^ CD4^+^ T_conv_ cells to eT_reg_ cells (also defined as PD-1^+^ CTLA-4^hi^) (**Figure 2F**). These results suggest that at homeostasis eT_reg_ cells receive increased constitutive TCR activation while experiencing ongoing SHP2 mediated restriction of these signals.

### Homeostatic blockade of PD-L1 and CTLA-4 enhances the eT_reg_ compartment

**Figure 3:**
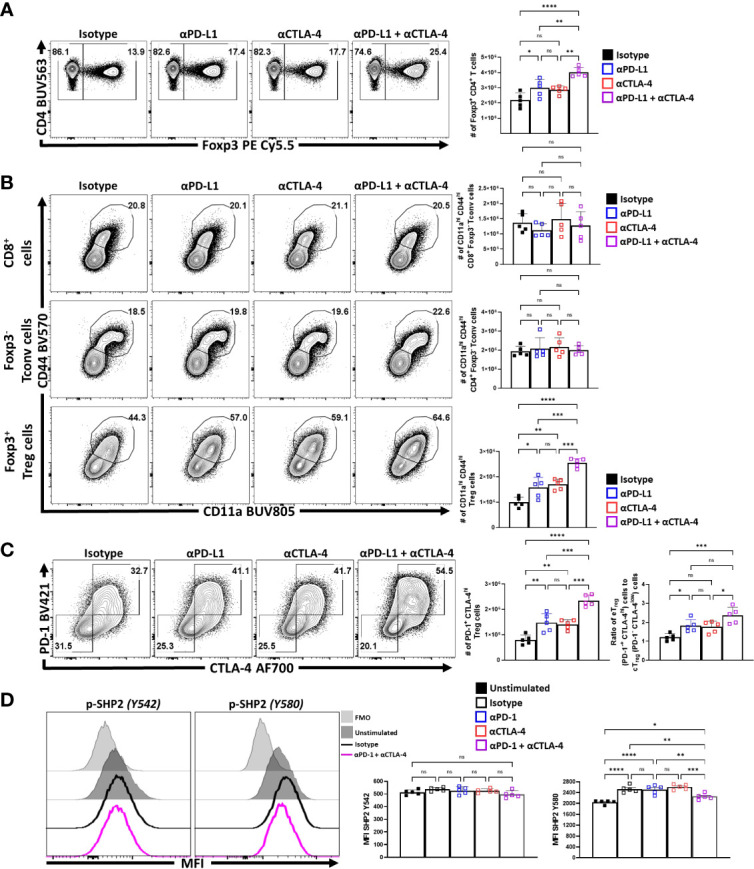
PD-1 and CTLA-4 additively restrict activated eT_reg_ cells at homeostasis. Cohorts of 8 week-old male C57BL/6 mice were given a single intraperitoneal injection of either αPD-L1, or αCTLA-4, or combination αPD-L1 and αCTLA-4, or Isotype control antibody. Splenocytes were harvested for analysis 72 hours later and analyzed via high parameter flow-cytometry. **(A)** Flow plots of bulk CD4^+^ T cells demonstrating increases in the proportion and number of T_reg_ cells following either blockade, with greatest enrichments occurring with combination blockade (n = 5/group, 1-way ANOVA with Tukey’s multiple comparisons test, *p < 0.05, **p < 0.01, ***p < 0.001, ****p < 0.0001, 3 experimental replicates). **(B)** Comparison of the proportions and number of CD44^hi^ CD11a^hi^ populations between CD8^+^ T_conv_, CD4^+^ T_conv_, and T_reg_ cells following 72 hours of single or combination α-PD-L1/CTLA-4 checkpoint blockade treatment (n = 5/group, 1-way ANOVA with Tukey’s multiple comparisons test, **p < 0.01, ***p < 0.001, ****p < 0.0001, 3 experimental replicates). **(C)** Flow plots of cT_reg_ (PD-1^-^ CTLA-4^low^), and eT_reg_ (PD-1^+^ CTLA-4^hi^) cells following blockade treatment, demonstrating enrichment of PD-1^+^ CTLA-4^hi^ cells with either blockade, with the greatest enrichment occurring when both pathways were blocked (n = 5/group, 1-way ANOVA with Tukey’s multiple comparisons test, **p < 0.01, ***p < 0.001, ****p < 0.0001, 3 experimental replicates), and subsequent ratio of eT_reg_ to cT_reg_ cells that were shifted with treatment (n = 5/group, 1-way ANOVA with Tukey’s multiple comparisons test, *p< 0.05, **p < 0.01, ***p < 0.001, ****p < 0.0001, 3 experimental replicates). **(D)** Enriched bulk CD4^+^ T cells were treated with either αPD-1, or αCTLA-4, or combination αPD-1 and αCTLA-4, or Isotype control antibody, and then stimulated with plate-bound α-CD3, PD-L1-Fc, and CD80-Fc and phospho-stained. Depicted are histogram comparisons of the T_reg_ subset (CD4^+^ Foxp3^+^) comparing gMFI of p-SHP2 at tyrosine residues Y542 and Y580 on T_reg_ cells (n = 5/group, 1-way ANOVA with Fisher’s LSD individual comparisons test, *p < 0.05, **p < 0.01, ***p < 0.001, ****p < 0.0001, 2 experimental replicates). All data presented are means +/- SD and show individual data points.

Previous studies showed that blockade of PD-L1 at homeostasis resulted in enhanced T_reg_ cell responses within three days (29) that was apparent for as long as 5 days (data not shown). To determine whether CTLA-4 also plays a similar role and how it relates to PD-1, cohorts of 8-week-old C57BL/6 mice were treated with a single intraperitoneal injection of control antibodies alone or in combination with α-PD-L1, α-CTLA-4, or a combination of α-PD-L1 and α-CTLA-4. Splenocytes from these hosts were harvested 72 hours later and analyzed *via* flow cytometry. The blockade of PD-L1 or CTLA-4 resulted in a significant enrichment in the proportion and total number of T_reg_ cells, yet when these blocking antibodies were combined there was an additive increase in the number of T_reg_ cells (**Figure 3A**). This was accompanied by a concurrent increase in the proportion and total number of activated (CD11a^hi^ CD44^hi^) eT_reg_-associated cells, which correlated with the observed total increase in T_reg_ cells (**Figure 3B**). This short-term blockade of the PD-1 and CTLA-4 pathways did not impact the non-T_reg_ subsets (CD4^+^ T_conv_, and CD8^+^ T cells) but resulted in increases in activated (CD11a^hi^ CD44^hi^) T_reg_ cells with further increases in the co-blockade treated hosts (**Figure 3B**). The enrichment of activated eT_reg_ cells correlated with increases in PD-1^+^ CTLA-4^hi^ T_reg_ cells with either blockade and when PD-1 and CTLA-4 were simultaneously blocked there was an additive increase in the ratio of eT_reg_ cells to cT_reg_ cells (**Figure 3C**).

Next, the impact of combination blockade on phosphorylation of suppressive SHP2 tyrosine phosphatases during activation was considered. SHP2 tyrosine phosphatase activity restricts CD28-mediated co-stimulation (32), and there are two tail tyrosine residues; Y542, which mitigates SHP2 phosphatase activity and Y580, which stimulates suppressive SHP2 phosphatase activity associated with signals from PD-1 and CTLA-4 (46). To evaluate whether PD-1 and CTLA-4 blockade would affect the immediate response to TCR associated SHP2 phosphorylation splenocyte-derived MACS enriched CD4^+^ T cells from naïve mice (**Supplemental Figure 2A**) were treated ex vivo with either an isotype control, α-PD-1, α-CTLA-4, or α-PD-1 plus α-CTLA-4. These cells were then transferred to plates coated with PD-L1-Fc, CD80-Fc, and α-CD3 in serum-free media. After incubating the cells for only 1 hour, to avoid complications associated with long term activation, the cells were fixed and phosphorylation of SHP2 tyrosine residues Y542 and Y580 were measured *via* flow cytometry. Firstly, T_reg_ cells stimulated with plate-bound PD-L1-Fc, CD80-Fc, and α-CD3, did not demonstrate any clear differences in the amount of phosphorylated SHP2 Y542 (pY542), but did have an increase in phosphorylated SHP2 Y580 (pY580) **(** **Figure 3D**). Interestingly, cells that were pre-treated with individual blockades of α-PD-1 or α-CTLA-4 did not yield any differences in the amount of pY580 observed but, when both PD-1 and CTLA-4 were blocked, the levels of pY542 remained constant but the amount of pY580 was significantly reduced (**Figure 3D**; **Supplemental Figure 2B**). These data sets indicate that for Treg cells that PD-1 and CTLA-4 can simultaneously contribute to the phosphorylation of TCR-suppressive Y580 that is independent of changes to the Y580-disabling Y542 residue.

**Figure 4:**
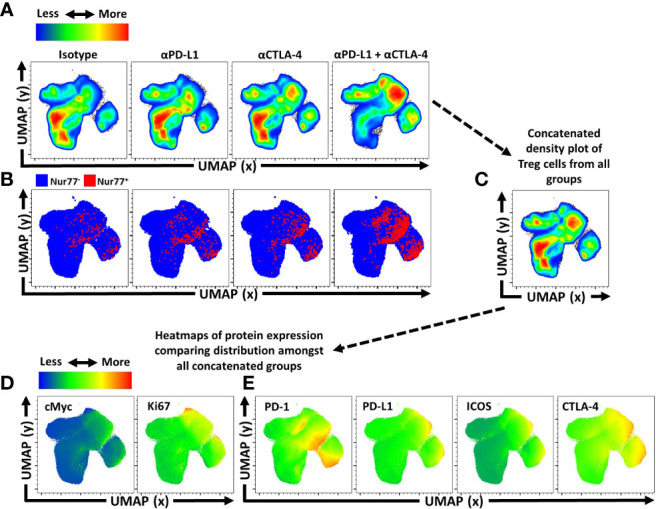
Activated and proliferative T_reg_ compartment phenotypic shifts following checkpoint blockade. Cohorts of 8 week-old male C57BL/6 mice were given a single intraperitoneal injection of either αPD-L1, or αCTLA-4, or combination αPD-L1 and αCTLA-4, or Isotype control antibody. Splenocytes were harvested for analysis 72 hours later and analyzed via high parameter flow-cytometry (3 experimental replicates). **(A)** Bulk T_reg_ sample data from each treatment group (n=5/group, 20 individuals total) was concatenated into a single sample and then evaluated using Uniform Manifold Approximation and Projection (UMAP) analysis (**Supplemental Figure 3A** for description) to produce 2-dimensional plots containing the measured parameters excluding PD-L1 and CTLA-4 from analysis to portray qualitative trends that emerged following treatment. **(A)** The individual UMAP analysis was then sub-divided into treatment specific UMAP sub-plots from the concatenated analysis depicting pseudo-color density distribution of T_reg_ cells amongst each treatment group within the UMAP. **(B)** Nur77^+^ cells (red) overlaid Nur77^-^ cells (blue) amongst the reference UMAP plots for each treatment group depicting enrichment of Nur77^+^ Treg cells with individual and combination blockade treatment. **(C)** Representation of the cumulative UMAP density plot depicting the assimilation of concatenated UMAP data from the 4 treated groups in figure **(A, D, E)** Median heatmap expression of proteins based on the total concatenated UMAP analysis, depicting expression of the protein labeled in each plot, allowing qualitative comparison to population density shifts demonstrated in **(A)**. **(D)** Heatmap expression of proliferation associated proteins cMyc and Ki67, with extensive overlap with enriched regions following individual or combination blockade, with more activated and proliferative cells accumulating in the upper right region of the UMAP plots, and more quiescent cells in the bottom left region of the UMAP plots. **(E)** Heatmap expression of B7-family costimulatory proteins, PD-1, PD-L1, ICOS, and CTLA-4, with enrichment in the regions correlating to blockade treatment.

Another approach to depict how these treatments impacted the T_reg_ cell populations was to utilize Uniform Manifold Approximation and Projection (UMAP) analysis of the concatenated data sets generated using an extensive panel of proteins expressed by T_reg_ cells from each of the treated groups, excluding the expression of PD-L1 and CTLA-4 from analytical algorithms (**Supplemental Figure 3A**). Following UMAP analysis, the samples were then unmixed into respective treatment groups and changes in distribution density within the UMAP analysis depicted across the different treatment groups (**Figure 4A**). Thus, comparison of the isotype treated with the combination treatment shows a marked shift in the heat map associated with expansion of eT_reg_ cells. The inclusion of staining for Nur77, a protein expressed proximally to TCR activation (47), allowed these events to be overlaid on respective UMAPs. This analysis illustrates how individual, or combination PD-L1/CTLA-4 blockade led to enrichment of Nur77 expression associated with eT_reg_ cells (**Figure 4B**). Then, using the original concatenated UMAP (**Figure 4C**), median fluorescence expression heatmaps were created to show comparative expression of proliferation-associated proteins (cMyc and Ki67) (**Figure 4D**), and co-stimulation associated proteins (**Figure 4E**). Compared to isotype treated hosts, the PD-L1 and CTLA-4 blockade treated hosts had increased enrichment in regions that overlap with Foxp3 and Helios, yet no clear enrichment over the CD25^hi^ regions of the UMAP while combination blockade hosts had even further enrichment over the Foxp3^hi^ and Helios^+^ regions and a comparative reduction of CD25^hi^ cells in addition to enrichment of CD73^hi^ T_reg_ cells (**Supplemental Figure 3B**). Likewise, either blockade resulted in enrichment in regions of the UMAP associated with activation (**Figure 4B**) or proliferation (**Figure 4D**), or expression of B7-family co-stimulation proteins (**Figure 4E**), with the greatest enrichments occurring in the cohort treated with the combination blockade.

**Figure 5:**
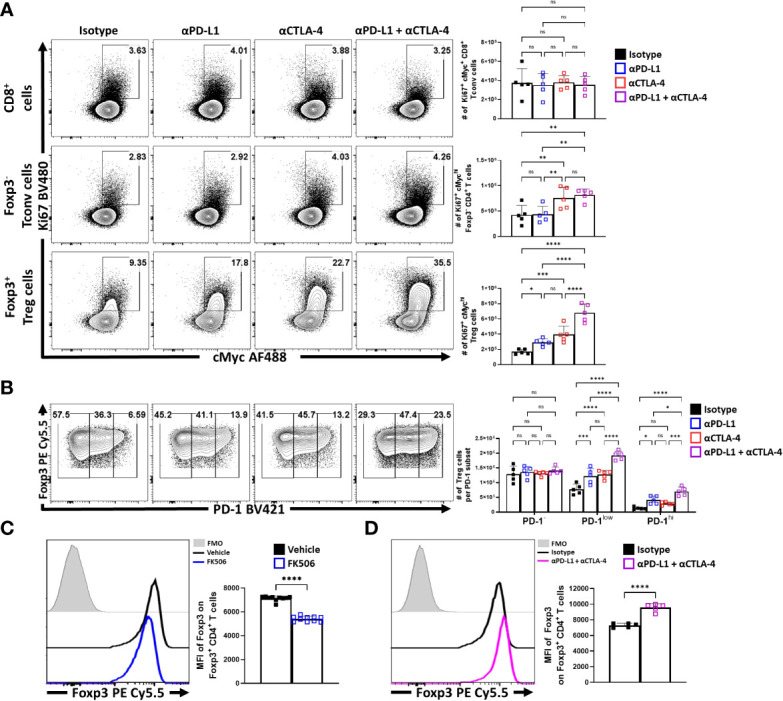
Blockade of PD-L1 and CTLA-4 additively drive enrichment and proliferation of the eT_reg_ compartment. Cohorts of 8 week-old male C57BL/6 mice were given a single intraperitoneal injection of either αPD-L1, or αCTLA-4, or combination αPD-L1 and αCTLA-4, or Isotype control antibody. Splenocytes were harvested for analysis 72 hours later and analyzed via high parameter flow-cytometry. **(A)** Flow plots comparing the proportion and number of Ki67^+^ cMyc^hi^ T cells cells following individual or combination PD-L1/CTLA-4 blockade treatment, subdivided into CD8^+^, CD4^+^ T_conv_, and T_reg_ cells (n = 5/group, 1-way ANOVA with Fisher’s LSD individual comparisons test, *p < 0.05, **p < 0.01, ***p < 0.001, ****p < 0.0001, 3 experimental replicates). **(B)** Flow plots comparing the proportions and number of PD-1^-^, PD-1^low^, and PD-1^hi^ T_reg_ cells following individual or combination blockade treatment, with enrichments occurring within the PD-1^+^ eT_reg_ associated subsets following blockade, the greatest of which occur with combination blockade treatment (2-way ANOVA with Sidak’s multiple comparisons test, *p < 0.05, ***p < 0.001, ****p < 0.0001, 3 experimental replicates). **(C)** Cohorts of 8 week-old male C57BL/6 mice were treated once daily for 4 days with subcutaneous injections of PBS/vehicle or Tacrolimus (FK506), and splenocytes were harvested and analyzed via flow cytometry. Comparative histograms of T_reg_ cells from vehicle control and FK506 treated mice demonstrating decreases in the gMFI of Foxp3 in T_reg_ cells in FK506 treated hosts (n = 10/group two-tailed unpaired student’s t-test, ****p < 0.0001, 2 experimental replicates). **(D)** Comparative histograms of T_reg_ cells from isotype and αPD-L1/αCTLA-4 combination blockade treated mice demonstrating increases in the gMFI of Foxp3 in T_reg_ cells from blockade treated hosts (n = 5/group two-tailed unpaired student’s t-test, ****p < 0.0001, 3 experimental replicates). All data presented are means +/- SD and show individual data points.

To compare the impact of inhibitory receptor blockade on the proliferative responses of conventional and T_reg_ cells, expression of Ki67 and cMyc was assessed. In these experiments, short term blockade did not lead to increased proliferation of CD8^+^ T cells (**Figure 5A**). For CD4^+^ T_conv_ cells, a modest increase in the percentage of proliferative cells (from 2 to 4%) was only observed with treatments that included α-CTLA-4. In contrast, T_reg_ cells demonstrated a marked increase of the Ki67^+^ cMyc^hi^ population with either PD-L1 or CTLA-4 blockade, with the most prominent increase observed when both were blocked (**Figure 5A**). This observation was consistent with the increased number of the PD-1^+^ T_reg_ subsets (PD-1^low^, PD-1^hi^) (**Figure 5B**). Additionally, TCR stimulation of T_reg_ cells is associated with maintenance of Foxp3 expression (10), and antagonism of TCR activity by treatment of mice for 4 days with tacrolimus (FK506) (48) resulted in a reduced MFI of Foxp3 in T_reg_ cells (**Figure 5C**). In contrast, the blockade of PD-L1 or CTLA-4 resulted in an overall increase in the MFI of Foxp3 amongst the bulk T_reg_ compartment, with the combination blockade having the greatest enhancement (**Figure 5D**; **Supplemental Figure 3C** for individual eT_reg_ blockade comparisons). Combined with the numerical, phenotypic and phos-data sets, these results highlight that PD-1 and CTLA-4 additively contribute to restrict the population of TCR-driven eT_reg_ cells.

To directly assess whether the enhanced eT_reg_ cell populations observed after blockade of PD-L1 and/or CTLA-4 at homeostasis would impact the ability to generate *de novo* T cell responses, these pathways were blocked in naïve mice that were then immunized with a non-replicative form of *Toxoplasma gondii* that expresses OVA. This vaccine strain provides a system to assess the activities required to generate effector T cell responses (42, 49). In these studies, mice were treated with isotype, α-PD-L1 or α-CTLA-4 and three days later were recipients of OTI T cells. A day later mice were vaccinated with CPS parasites and then re-treated with the relevant antibodies. Seven days post-vaccination mice were assessed for T_reg_ cell populations, parasite specific CD4^+^ T cell responses as well the OTI T cells. At this time point (11 days after initial treatment), enhanced eT_reg_ cell responses were still obvious, indicating that the effects of CTLA-4 and PD-L1 blockade were sustained (**Supplemental Figures 5A, B**). However, despite this enhanced eT_reg_ cell activity the magnitude of the CPS-induced T cell responses were not reduced (**Supplemental Figures 5C, D**) but they did profoundly skew the eT_reg_ to T_conv_ T_eff_ ratios (**Supplemental Figures 5E, F**). However, it is relevant to note that in these experiments the use of α-CTLA-4 alone resulted in heightened OTI and endogenous CD4^+^ T cell responses (**Supplemental Figures 5C, D**) but this was antagonized by the inclusion of anti-PD-L1. This antagonism of the T cell responses correlated with conditions that resulted in the presence of the highest numbers of eTreg cells across multiple experiments.

**Figure 6:**
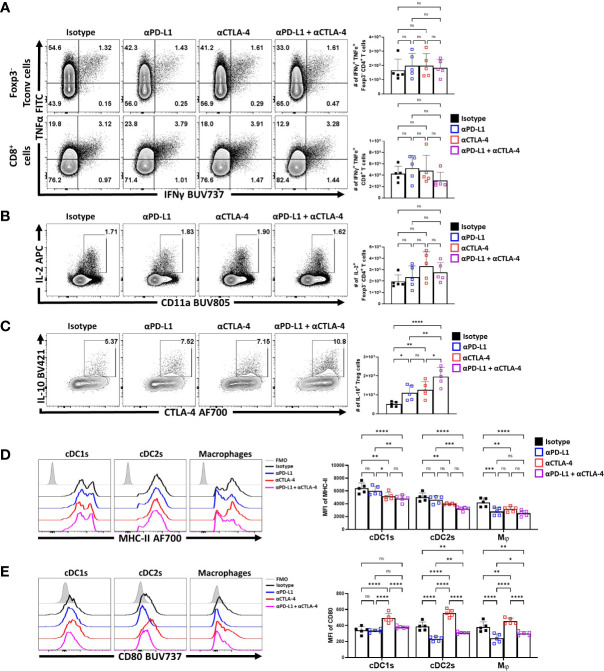
Combo-blockade of PD-L1 and CTLA-4 drives a myeloid-suppressive T_reg_ environment. 8 week-old male C57BL/6 mice were given a single intraperitoneal injection of either αPD-L1, or αCTLA-4, or combination αPD-L1 and αCTLA-4, or Isotype control antibody. At 72 hours following treatment, their splenocytes were harvested, and stimulated with PMA/Ionomycin for cytokine staining and analyzed via flow cytometry. **(A)** Plots depicting the expression of IFNγ and TNFα on CD4^+^ T_conv_ and CD8^+^ cells from single and combo blockade treated hosts (n = 5/group, 1-way ANOVA with Fisher’s LSD individual comparisons test, 2 experimental replicates). **(B)** Plots depicting the expression of IL-2 on CD4^+^ T_conv_ cells from single and combo blockade treated hosts (n = 5/group, 1-way ANOVA with Fisher’s LSD individual comparisons test, 2 experimental replicates). **(C)** Plots depicting the expression of IL-10 and CTLA-4 on bulk T_reg_ cells from each treatment group, with increases in IL-10^+^ CTLA-4^hi^ T_reg_ cells from single and combo blockade treated hosts (n = 5/group, 1-way ANOVA with Fisher’s LSD individual comparisons test, *p < 0.05, **p < 0.01, ****p < 0.0001, 2 experimental replicates). **(D)** Ex-vivo staining of splenocytes evaluating the expression of MHC-II on cDC1s (CD3^-^, B220^-^, CD19^-^, NK1.1^-^, Ly6G^-^, CD64^-^, CD11c^+^, MHC-II^+^, XCR1^+^), cDC2s (CD3^-^, B220^-^, CD19^-^, NK1.1^-^, Ly6G^-^, CD64^-^, CD11c^+^, MHC-II^+^, SIRPα^+^), and macrophages (CD3^-^, B220^-^, CD19^-^, NK1.1^-^, Ly6G^-^, CD64^+^, CD11b^+^, MHC-II^+^, Ly6C^low^) (**Supplemental Figure 4** for description) following blockade treatments, with decreasing trends MHC-II with combo blockade (n = 5/group, 2-way ANOVA with Fisher’s LSD individual comparisons test, *p < 0.05, **p < 0.01, ***p < 0.001, ****p < 0.0001, 3 experimental replicates) **(E)** Plots comparing CD80 expression on cDCs and Macrophages, demonstrating changes to surface CD80 based on blockade treatment (n = 5/group, 2-way ANOVA with Fisher’s LSD individual comparisons test, *p < 0.05, **p < 0.01, ****p < 0.0001, 3 experimental replicates) All data presented are means +/- SD and show individual data points.

Nevertheless, to further assess the impact of IR blockade on conventional T cells and T_reg_ cell function at homeostasis, splenocytes from naïve treated hosts were stimulated with PMA and ionomycin and the ability to produce cytokines was assessed. The Foxp3^-^ CD4^+^ and CD8^+^ T cells readily produced TNFα, and there was a small proportion of these cells that co-expressed TNFα and IFNγ. Following solo, or combined PD-L1 and CTLA-4 blockade there were no significant increases in the production of these cytokines **(** **Figure 6A**). Likewise, a small proportion of the CD4^+^ T cell population produces IL-2, but this was not altered by these treatments (**Figure 6B**). Thus, consistent with the data in **Figure 4**, these short term blockades did not appear to lead to any obvious enhancement of the incipient T cell response or levels of IL-2 that might contribute to the enhanced eT_reg_ population observed.

T_reg_ cell production of IL-10 is one important function of these cells, and this cytokine can act on APCs and limit their expression of MHC class II and CD80. In contrast, the ability of CTLA-4 to bind to and strip CD80 from these cells can reduce costimulation (34). In these *in vivo* studies, blockade of PD-L1 or CTLA-4 resulted in an increase in the number of IL-10^+^ T_reg_ cells, with the combination blockade resulting in the greatest increase (**Figure 6C**). Evaluation of the splenic DC and macrophages compartments ex vivo (**Supplemental Figure 4**) showed that cDC2s and macrophages had varied expression of MHC class II and CD80. The blockade of PD-L1 alone resulted in modest reductions in MHC-II expression, particularly amongst macrophages (**Figure 6D**). Comparatively, solo CTLA-4 blockade drove reductions in MHC-II particularly on DCs while the combined blockade of both PD-L1 and CTLA-4 had consistent trends of decreasing MHC class II expression in cDCs and macrophages (**Figure 6D**). In context of co-stimulatory CD80, PD-L1 blockade alone reduced CD80 on cDC2s and macrophages, but not cDC1s (**Figure 6E**). In comparison and consistent with the ability of CTLA-4 to strip CD80 (34), CTLA-4 blockade resulted in increased CD80 expression on cDCs and macrophages (**Figure 6E**). When blockade treatments were combined, the effects of anti-PD-L1 were dominant with reduction in the expression of CD80. This result established that not only do these treatments favor the expansion of the eT_reg_ compartment, but this correlates with reduced APC functions of other cell types that are known to be impacted by T_reg_ cells.

## Discussion

The focus on the role of PD-1 and CTLA-4 in limiting effector T cell responses has revealed that the expression of these molecules is a byproduct of repeated TCR stimulation over time (50–52). The studies presented here focus on the impact of these potentially overlapping pathways on T_reg_ cell homeostasis and in particular on the differences between eT_reg_ and cT_reg_ cell populations. In this context, short term homeostatic blockade of PD-L1 or CTLA-4 did not result in appreciable activation of T_conv_ CD4^+^ or CD8^+^ T cells. Subsequent evaluation of the possible impact of these enhanced eT_reg_ populations on the formation of T cell responses to vaccination with OVA-expressing parasites did not antagonize the expansion of transferred OTI populations or endogenous effector CD4^+^ T cells during this challenge. However, these vaccination studies do not distinguish the effects of blockade of CTLA-4 or PD-L1 on the T_reg_ cell populations versus an impact on the expansion of the parasite specific effectors. For example, CTLA-4 blockade treatment alone led to an expansion in the formation of effector T cell responses. However, the observation that CTLA-4 blockade in combination with PD-L1 blockade treatment resulted in an even greater expansion of eT_reg_ cells and antagonized the effects of solo CTLA-4 blockade on endogenous and effector T cells and transferred OTI cells, of which would be consistent with a role for these heightened eT_reg_ populations to limit effector responses. Nevertheless, these studies need to be interpreted with care and additional studies that allow the isolation of the effects of PD-1 and CTLA-4 on activated CD4^+^ and CD8^+^ T cells versus T_reg_ cells in the same environment would be required.

In considering these findings, T_reg_ cells receive ongoing TCR signaling which is required to maintain expression of Foxp3 and their suppressive capacity (10). When PD-1 and CTLA-4 signaling is mitigated, there is increased overall expression of Foxp3 on T_reg_ cells, which is directly contrasted by short-term blockade of TCR activation which results in reduced T_reg_ Foxp3 expression. Earlier reports suggested that PD-1 and CTLA-4 are associated with the suppressive functions of T_reg_ cells (53, 54), but a consensus is emerging that these inhibitory receptors can individually restrict T_reg_ capacity and suppressive function during autoimmune disease (20, 28), cancer (21, 41, 55) and infection (29). Thus, co-blockade of PD-L1 and CTLA-4 resulted in increased T_reg_ cell proliferation, percentage (but not relative levels) of cells that produced IL-10 and expressed CD73, CTLA-4, and PD-L1, and a reduction in markers of APC activation. Together, these results suggest that targeting PD-1 and CTLA-4 does not result in an increase in T_reg_ suppressive activity per se, but rather that these pathways act to limit the size of the effector T_reg_ pool.

We now appreciate that while the cT_reg_ compartment makes greater use of STAT5 signaling cytokines such as IL-2 for maintenance (11, 13, 56) the eT_reg_ compartment is more dependent on TCR-mediated activation and co-stimulation to survive. This is suggested by the finding that the eT_reg_ subset has higher basal levels of pZAP70, pAKT, and pmTOR, than the cT_reg_ compartment. However, eT_reg_ cells also express PD-1 which interacts with SHP2 to antagonize T cell activation (57). In comparing PD-1 to CTLA-4 which is also expressed on eT_reg_ cells, PD-1 has been directly implicated in binding SHP2, while CTLA-4 is missing a motif that would allow recognition of SHP2, but CTLA-4 does function as a negative regulator of T cell activation (31, 40, 58). SHP2 associates with CTLA-4 and the TCR (59, 60) and Schneider & Rudd, (2000) postulated that this activity is mediated via its impact on PI3K, of which CTLA-4 signaling does have impacts on PI3K. Notably, CTLA-4 does associate with SHP2 in T cells, and possibly has indirect interactions with SHP2 mediated by an intermediate which is still unclear (31). Here, there is an observation that the eT_reg_ subset may have an enhanced capacity to respond to TCR signals while simultaneously being sensitive to negative SHP2-associated signals from PD-1 or CTLA-4.

There are multiple possible mechanisms whereby blockade of these IR may lead to enhanced Treg cell activities. Several imaging studies have highlighted that when compared to activated CD4^+^ T cells, T_reg_ cell interactions with DC are characterized by less stable short term contacts (61, 62), and a recent report highlighted that T_reg_ cell use CTLA-4 to disrupt these interactions (41). Whether blockade of CTLA-4 leads to enhanced DC-Treg interactions remains to be tested. Likewise, previous studies have deployed strategies to evaluate the impact of SHP2 *via* T cell specific SHP2^-/-^ mice and highlighted that this pathway is redundant in exhaustion (63). However, in that report, during LCMV infection the loss of SHP2 resulted in enhanced expansion (almost 3 fold) of virus-specific effector CD8^+^ T cells and in a tumor model the ability of PD-1 blockade to enhance the percentage of total and IFNγ positive intra-tumoral CD8^+^ T cells was SHP2-dependent. While there may be SHP2-independent pathways that contribute to the activities of PD-1, these data sets remain consistent with the idea that PD-1 mediated engagement of SHP2 limits T cell activation. Indeed, this is reflected in our own data sets in which eT_reg_ cells had enhanced levels of pSHP2 and that mitigation of both PD-1 and CTLA-4 signaling pathways reduces suppressive pSHP2-Y580, which correlated with increased Foxp3 expression and numbers of eT_reg_ cells. Given the ubiquitous expression of SHP2 by cells of the immune system, whether this reduction in SHP2 activity in eT_reg_ cells accounts for their expansion will require the use of lineage-specific approaches to directly address this question.

While PD-1 and CTLA-4 are related B7 family members, engage SHP2 signaling, and seem to additively limit eT_reg_ proliferation and function, the current literature indicate that there is still distinction to their suppressive mechanisms. For example, PD-1 accumulates on the cell surface and is accessible to PD-L1 ligation (64). and thereby act in cis to limit T cell activation. For T_reg_ cells, blockade of this pathway resulted in enhanced numbers and IL-10 production and was associated with reduced APC expression of CD80 and MHC class II. In contrast, the majority of CTLA-4 is stored intracellularly and is translocated to the surface upon TCR stimulation (65, 66) where it can provide negative costimulatory signals (67). In addition, the ability of CTLA-4 to bind with high affinity to CD80 means that it can outcompete the ability of CD28 to provide costimulation and can actively restrict APC function through CTLA-4 mediated trogocytosis of CD80 (34). Thus, CTLA-4 is an invoked off switch which can act in cis and trans to limit eT_reg_ cells. Interestingly, this complex biology is apparent in the studies presented here: α-PD-L1 treatment alone drove a reduction in CD80 expression by cDC2s and macrophages, while α-CTLA-4 treatment still drove an enrichment of T_reg_ cells yet resulted in a significant increase in myeloid expression of CD80 (consistent with reduced trogocytosis). Nevertheless, that combination blockade of PD-L1 and CTLA-4 resulted in a reduction of myeloid CD80 expression suggests that the increased number of T_reg_ cells and their production of IL-10 is sufficient to exceed the effects of CTLA4 on CD80 levels.

The past twenty years has witnessed an increased utilization of immunotherapeutic drugs to enhance immune mediated control of certain cancers or to limit autoimmune inflammation. The blockade of PD-1 or CTLA-4 or the use of CTLA4-Ig are all examples of clinical interventions to impact effector T cell responses that are directly relevant to eT_reg_ cells and the pathways that we show here. However, these treatment strategies do not always prove effective, and their impact of T_reg_ cells may in part explain some of this heterogeneity in clinical outcome (21, 68, 69). Perhaps, the ability to specifically target these pathways (either to agonize or block) on eT_reg_ cells can be used as an immunotherapeutic strategy to enhance T_reg_ function to treat immunopathological diseases or select against T_reg_ mediated suppression in the context of infection or cancer.

## Data availability statement

The original contributions presented in the study are included in the article/**Supplementary Material**. Further inquiries can be directed to the corresponding author.

## Ethics statement

The animal study was reviewed and approved by University of Pennsylvania Institutional Animal Care and Use Committee.

## Author contributions

JP conceptualized the project, designed/executed all experiments, performed data analysis, figure production, and authored the paper. ZL, JC, AH, BD, LS and KO aided in data collection, provided conceptual feedback regarding experimental design, data analysis, and manuscript editing. DC directly supervised experimental execution, interpretation, and presentation of data. CH supervised the project in entirety. All authors contributed to the article and approved the submitted version.

## Supplementary material

Supplementary Figure 1
*T_reg_ subsetting.* **(A)** Flow cytometry sub-gating example strategy identifying T_reg_ cells, utilizing splenocytes from an 8 week-old male C57BL/6 mouse. **(B)** Gating strategy to identify PD-1^+^ CTLA-4^hi^ (eT_reg_) *vs* PD-1^-^ CTLA-4^low^ (cT_reg_) subsets. **(C)** Splenocytes from an 8 week-old and 16 week-old C57BL/6 mice were evaluated for their proportions of PD-1^+^ CTLA-4^hi^ eT_reg_ cells *(two-tailed unpaired student’s t-test, ** = p < 0.01). All data presented are means +/- SD and show individual data points.*

Supplementary Figure 2
*CD4 T cell enrichment and SHP2 phosphorylation results.* **(A)** Flow plots of from 8 week-old male C57BL/6 mouse splenocytes assessing CD3^+^ CD4^+^ T cell proportions following MACS enrichment *(two-tailed paired student’s t-test, **** = p < 0.0001, 2 experimental replicates).* **(B)** Enriched bulk CD4^+^ T cells were treated with either αPD-1, or αCTLA-4, or combination αPD-1 and αCTLA-4, or Isotype control antibody, and then stimulated with plate-bound α-CD3, PD-L1-Fc, and CD80-Fc and phospho-stained. Depicted are histogram comparisons of the T_reg_ subset (CD4^+^ Foxp3^+^) comparing gMFI of p-SHP2 at tyrosine residues Y542 and Y580 on T_reg_ cells *(n = 5/group, 1-way ANOVA with Fisher’s LSD individual comparisons test, * = p < 0.05, ** = p < 0.01, *** = p < 0.001, **** = p < 0.0001, 2 experimental replicates)*. *All data presented are means +/- SD and show individual data points.*

Supplementary Figure 3
*UMAP and eT_reg_ Foxp3 MFI following checkpoint blockade.* **(A)** Flow cytometry sub-gating example strategy identifying T_reg_ cells, utilizing splenocytes from an 8 week-old male C57BL/6 mouse. The UMAP was then generated using concatenated T_reg_ cells from 8 week-old male C57BL/6 mice that were given a single intraperitoneal injection of either αPD-L1, or αCTLA-4, or combination αPD-L1 and αCTLA-4, or Isotype control antibody. The calculated fluorescence factors that generated the UMAP are depicted. **(B)** Expression trends of T_reg_-associated proteins Foxp3, Helios, CD25, CD73, and CD122 amongst the T_reg_ compartment within the concatenated UMAP from **Figure 4C**. **(C)** Comparative histograms depicting the gMFI of Foxp3 on PD-1^+^ CTLA-4^hi^ eT_reg_ cells following checkpoint blockade *(n = 5/group, 1-way ANOVA with Fisher’s LSD individual comparisons test, * = p < 0.05, ** = p < 0.01, **** = p < 0.0001, 2 experimental replicates)*. *All data presented are means +/- SD and show individual data points.*

Supplementary Figure 4
*Myeloid Gating.* **(A)** Splenocytes from 8 week-old male C57BL/6 mice were analyzed *via* flow cytometry across multiple leukocyte populations as depicted: B cells (CD3^-^, B220^+^, CD19^+^, MHC-II^+^), cDC1s (CD3^-^, B220^-^, CD19^-^, NK1.1^-^, Ly6G^-^, CD64^-^, CD11c^+^, MHC-II^+^, XCR1^+^), cDC2s (CD3^-^, B220^-^, CD19^-^, NK1.1^-^, Ly6G^-^, CD64^-^, CD11c^+^, MHC-II^+^, SIRPα^+^), and macrophages (CD3^-^, B220^-^, CD19^-^, NK1.1^-^, Ly6G^-^, CD64^+^, CD11b^+^, MHC-II^+^, Ly6C^low^).

Supplementary Figure 5 Combination blockade does not enhance the formation of T cell immune responses to vaccination, instead results in an increase in the ratio of eT_reg_s: T_eff_ cells. 8 week-old male C57BL/6 mice were given a single intraperitoneal injection of either αPD-L1, or αCTLA-4, or combination αPD-L1 and αCTLA-4, or Isotype control antibody. At 72 hours following treatment, congenically labeled OTI cells were transferred IP, and the hosts were vaccinated with CPS-OVA 24 hours after OTI transfer. The mice were re-dosed with their respective blocking antibody another 24 hours after vaccination. At day 7 following vaccination, the spleens, peritoneal exudate cells (PEC), and draining lymph nodes (dLN) were harvested, and analyzed via flow cytometry. **(A)** Representative plots of splenocyte-derived bulk CD4^+^ T cells depicting the Foxp3^+^ subset from each blockade treatment group and statistically from each tissue harvested (n = 4-5/group, 1-way ANOVA with Fisher’s LSD individual comparisons test, * = p < 0.05, ** = p < 0.01, *** = p < 0.001, **** = p < 0.0001, 1 experimental replicate). **(B)** Representative plots of splenocyte-derived bulk Foxp3^+^ T_reg_ cells comparing the number of PD-1^+^ CTLA-4^hi^ eT_reg_ cells between blockade treatment groups and across each tissue evaluated (n = 4-5/group, 1-way ANOVA with Fisher’s LSD individual comparisons test, * = p < 0.05, ** = p < 0.01, *** = p < 0.001, **** = p < 0.0001, 1 experimental replicate). **(C)** Representative plots of splenocyte-derived transferred OTI T cells (CD45.1^+^ CD8^+^) sub-gated on Tbet^+^ CD11a^hi^ populations following vaccination (n = 4-5/group, 1-way ANOVA with Fisher’s LSD individual comparisons test, * = p < 0.05, 1 experimental replicate) **(D)** Splenocyte-derived endogenous (CD45.2^+^) parasite-specific CD4^+^ T_conv_ effector T cell (T_eff_) responses (Tetramer^+^ Tbet^+^) to vaccination (n = 4-5/group, 1-way ANOVA Fisher’s LSD individual comparisons test, * = p < 0.05, ** = p < 0.01, ** = p < 0.01, 1 experimental replicate). **(E, F)** Graphed ratios of splenic eT_reg_ cells (PD-1^+^ CTLA-4^hi^) to transferred OTI T_eff_ cells (CD8^+^ CD45.1^+^ Tbet^+^ CD11a^hi^) (Left), and eT_reg_ cells to endogenous parasite-specific CD4^+^ T_conv_ effector T cell responses (Right) (n = 4-5/group, 1-way ANOVA Fisher’s LSD individual comparisons test, * = p < 0.05, 1 experimental replicate). All data presented are means +/- SD and show individual data points.
