# Supplementary figures and images for: PD-1 and CTLA-4 exert additive control of effector regulatory T cells at homeostasis
Source: Front Immunol. 2023 Mar 7;14:997376. doi: 10.3389/fimmu.2023.997376 (PMC10028286; doi:10.3389/fimmu.2023.997376)

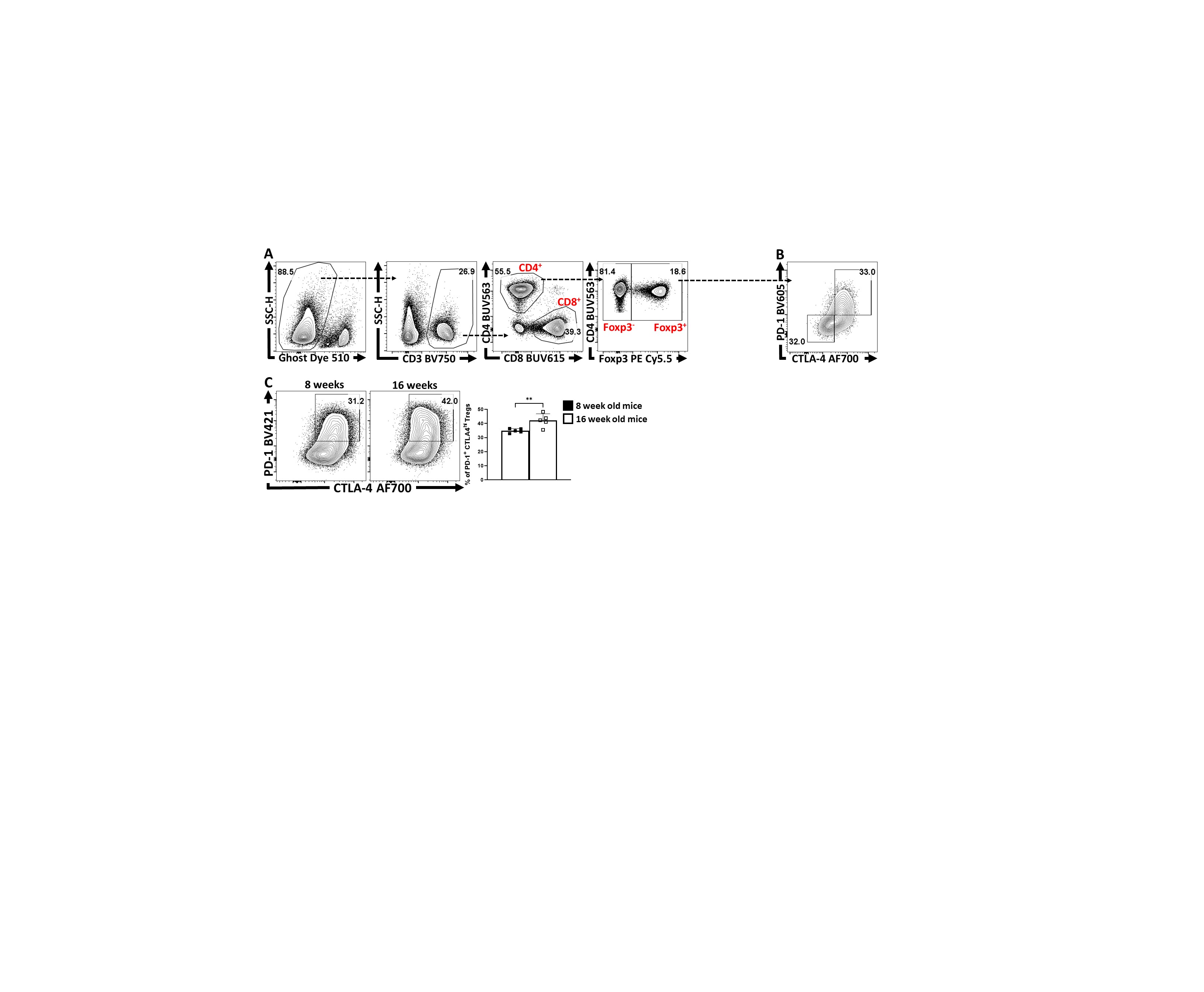

Supplement: Supplementary Figure 1 — Treg subsetting. (A) Flow cytometry sub-gating example strategy identifying Treg cells, utilizing splenocytes from an 8 week-old male C57BL/6 mouse. (B) Gating strategy to identify PD-1+ CTLA-4hi (eTreg) vs PD-1- CTLA-4low (cTreg) subsets. (C) Splenocytes from an 8 week-old and 16 week-old C57BL/6 mice were evaluated for their proportions of PD-1+ CTLA-4hi eTreg cells (two-tailed unpaired student’s t-test, ** = p < 0.01). All data presented are means +/- SD and show individual data points. [file Image_1.jpg]

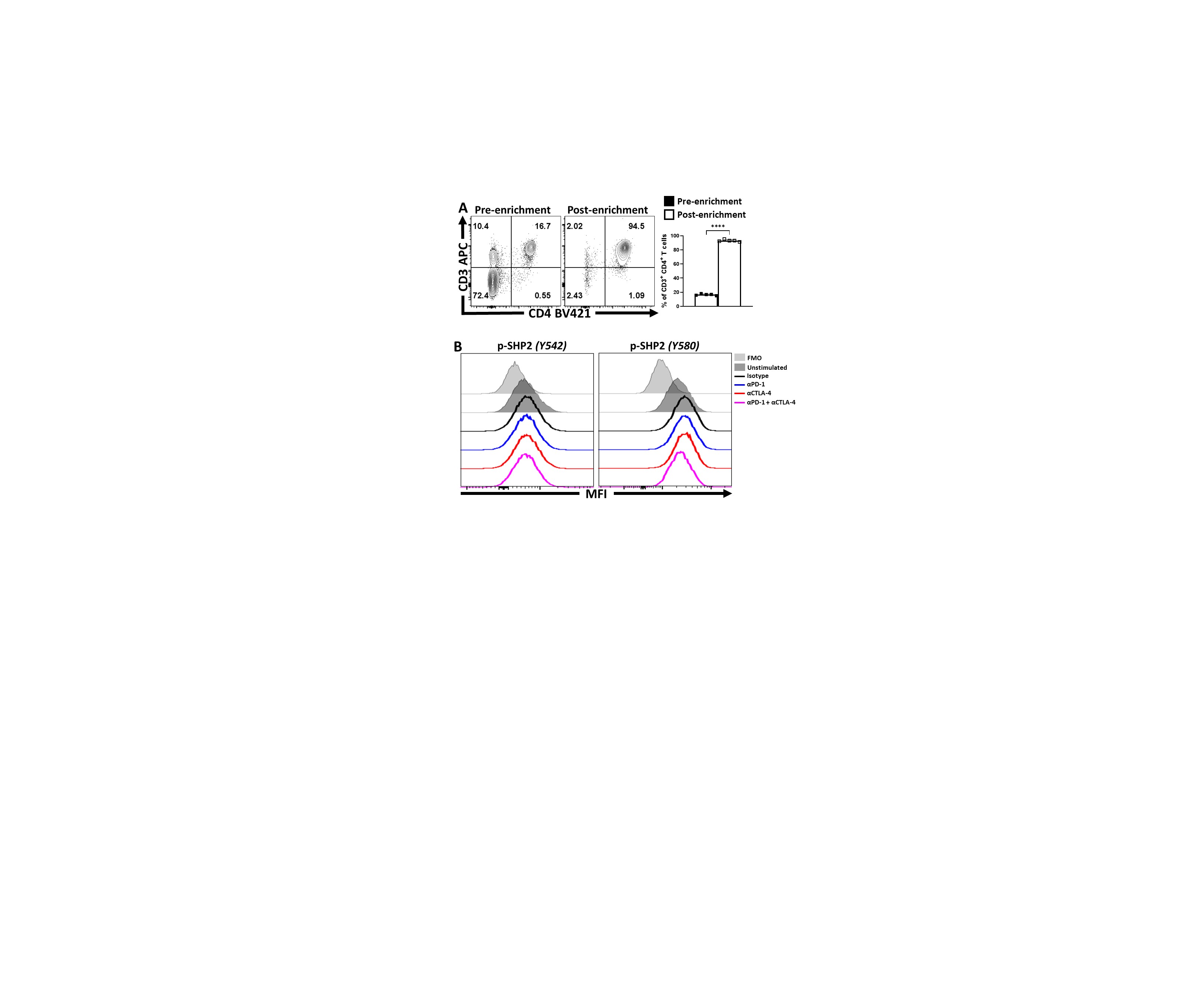

Supplement: Supplementary Figure 2 — CD4 T cell enrichment and SHP2 phosphorylation results. (A) Flow plots of from 8 week-old male C57BL/6 mouse splenocytes assessing CD3+ CD4+ T cell proportions following MACS enrichment (two-tailed paired student’s t-test, **** = p < 0.0001, 2 experimental replicates). (B) Enriched bulk CD4+ T cells were treated with either αPD-1, or αCTLA-4, or combination αPD-1 and αCTLA-4, or Isotype control antibody, and then stimulated with plate-bound α-CD3, PD-L1-Fc, and CD80-Fc and phospho-stained. Depicted are histogram comparisons of the Treg subset (CD4+ Foxp3+) comparing gMFI of p-SHP2 at tyrosine residues Y542 and Y580 on Treg cells (n = 5/group, 1-way ANOVA with Fisher’s LSD individual comparisons test, * = p < 0.05, ** = p < 0.01, *** = p < 0.001, **** = p < 0.0001, 2 experimental replicates). All data presented are means +/- SD and show individual data points. [file Image_2.jpg]

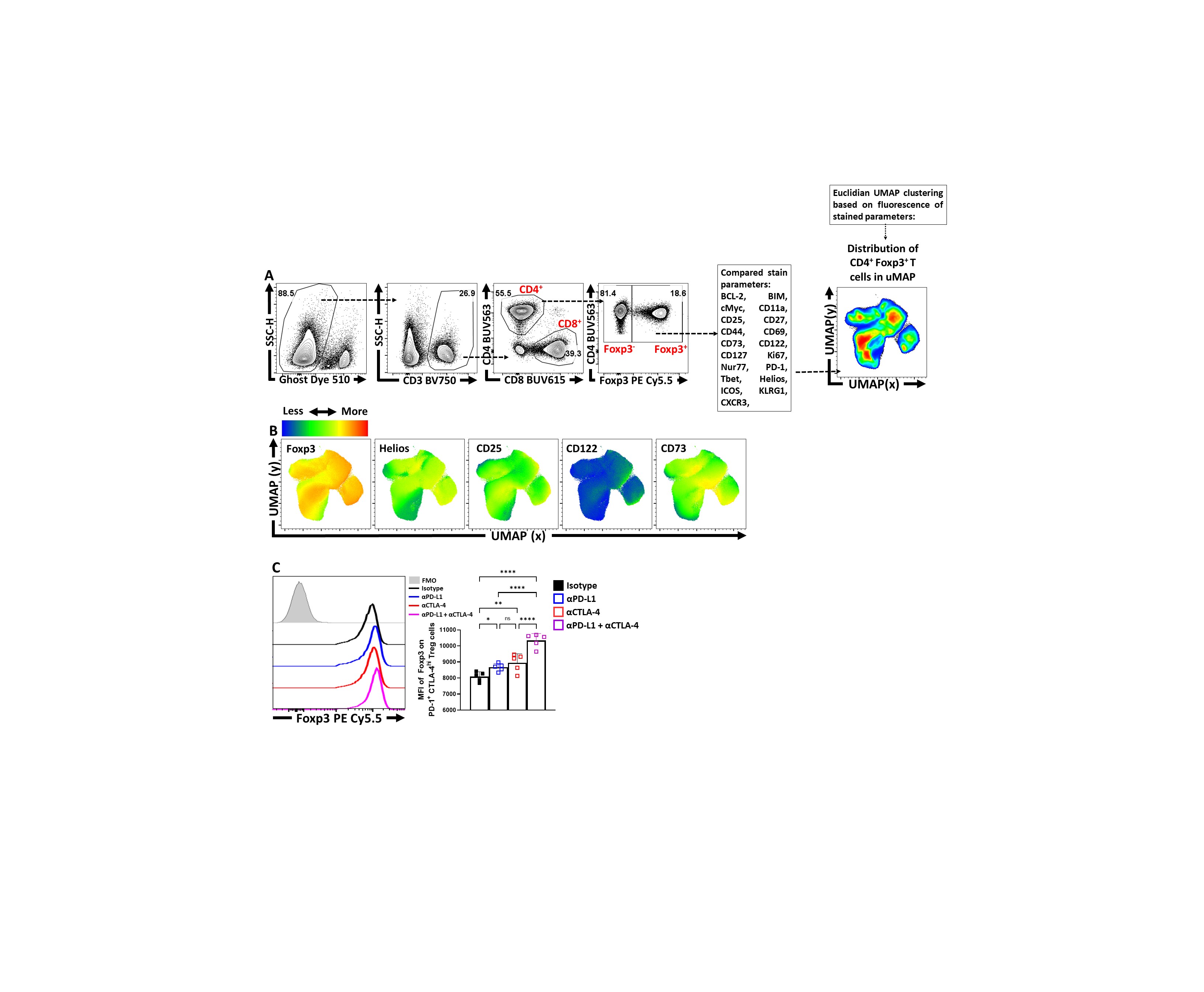

Supplement: Supplementary Figure 3 — UMAP and eTreg Foxp3 MFI following checkpoint blockade. (A) Flow cytometry sub-gating example strategy identifying Treg cells, utilizing splenocytes from an 8 week-old male C57BL/6 mouse. The UMAP was then generated using concatenated Treg cells from 8 week-old male C57BL/6 mice that were given a single intraperitoneal injection of either αPD-L1, or αCTLA-4, or combination αPD-L1 and αCTLA-4, or Isotype control antibody. The calculated fluorescence factors that generated the UMAP are depicted. (B) Expression trends of Treg-associated proteins Foxp3, Helios, CD25, CD73, and CD122 amongst the Treg compartment within the concatenated UMAP from Figure 4C . (C) Comparative histograms depicting the gMFI of Foxp3 on PD-1+ CTLA-4hi eTreg cells following checkpoint blockade (n = 5/group, 1-way ANOVA with Fisher’s LSD individual comparisons test, * = p < 0.05, ** = p < 0.01, **** = p < 0.0001, 2 experimental replicates). All data presented are means +/- SD and show individual data points. [file Image_3.jpg]

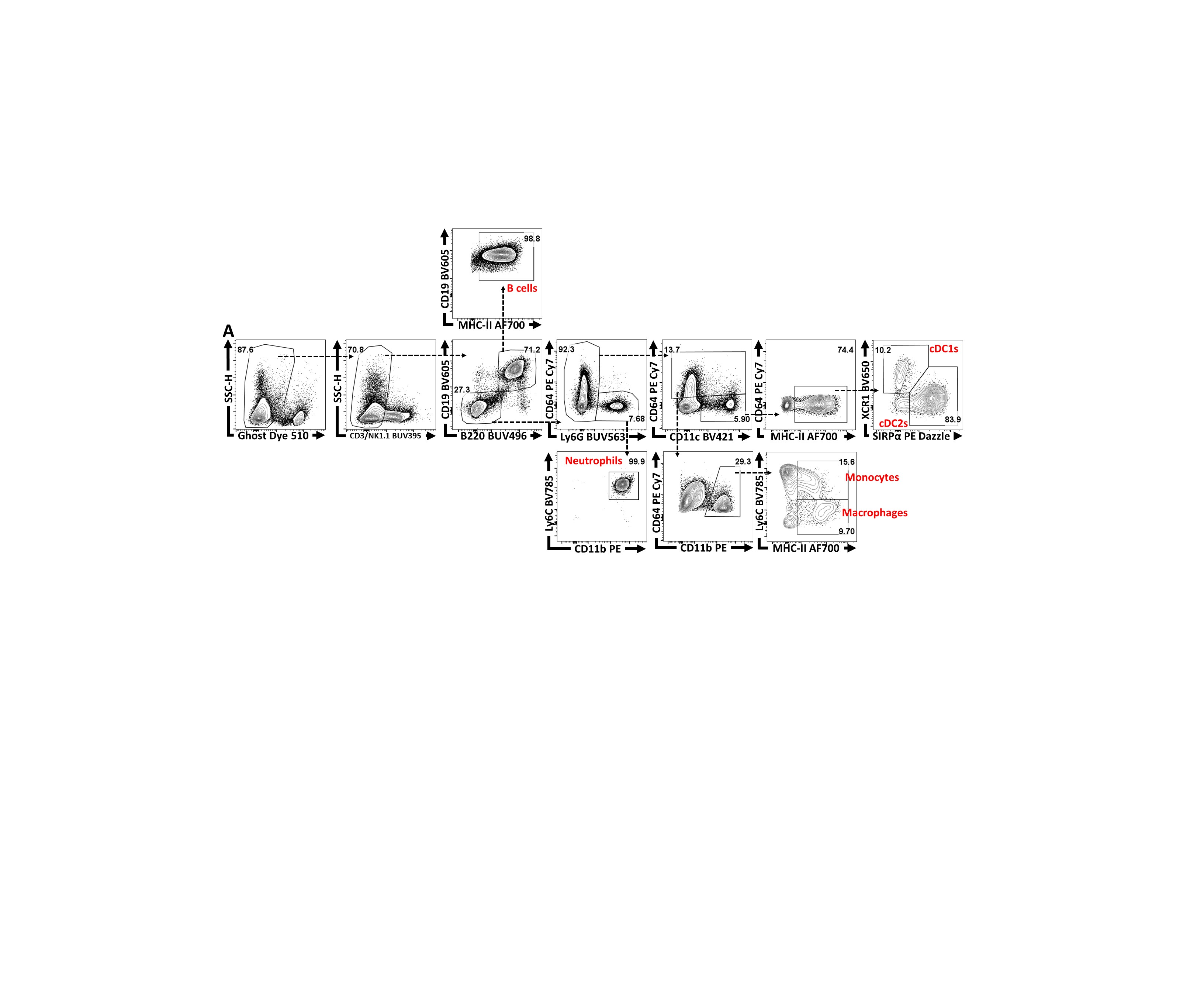

Supplement: Supplementary Figure 4 — Myeloid Gating. (A) Splenocytes from 8 week-old male C57BL/6 mice were analyzed via flow cytometry across multiple leukocyte populations as depicted: B cells (CD3-, B220+, CD19+, MHC-II+), cDC1s (CD3-, B220-, CD19-, NK1.1-, Ly6G-, CD64-, CD11c+, MHC-II+, XCR1+), cDC2s (CD3-, B220-, CD19-, NK1.1-, Ly6G-, CD64-, CD11c+, MHC-II+, SIRPα+), and macrophages (CD3-, B220-, CD19-, NK1.1-, Ly6G-, CD64+, CD11b+, MHC-II+, Ly6Clow). [file Image_4.jpg]

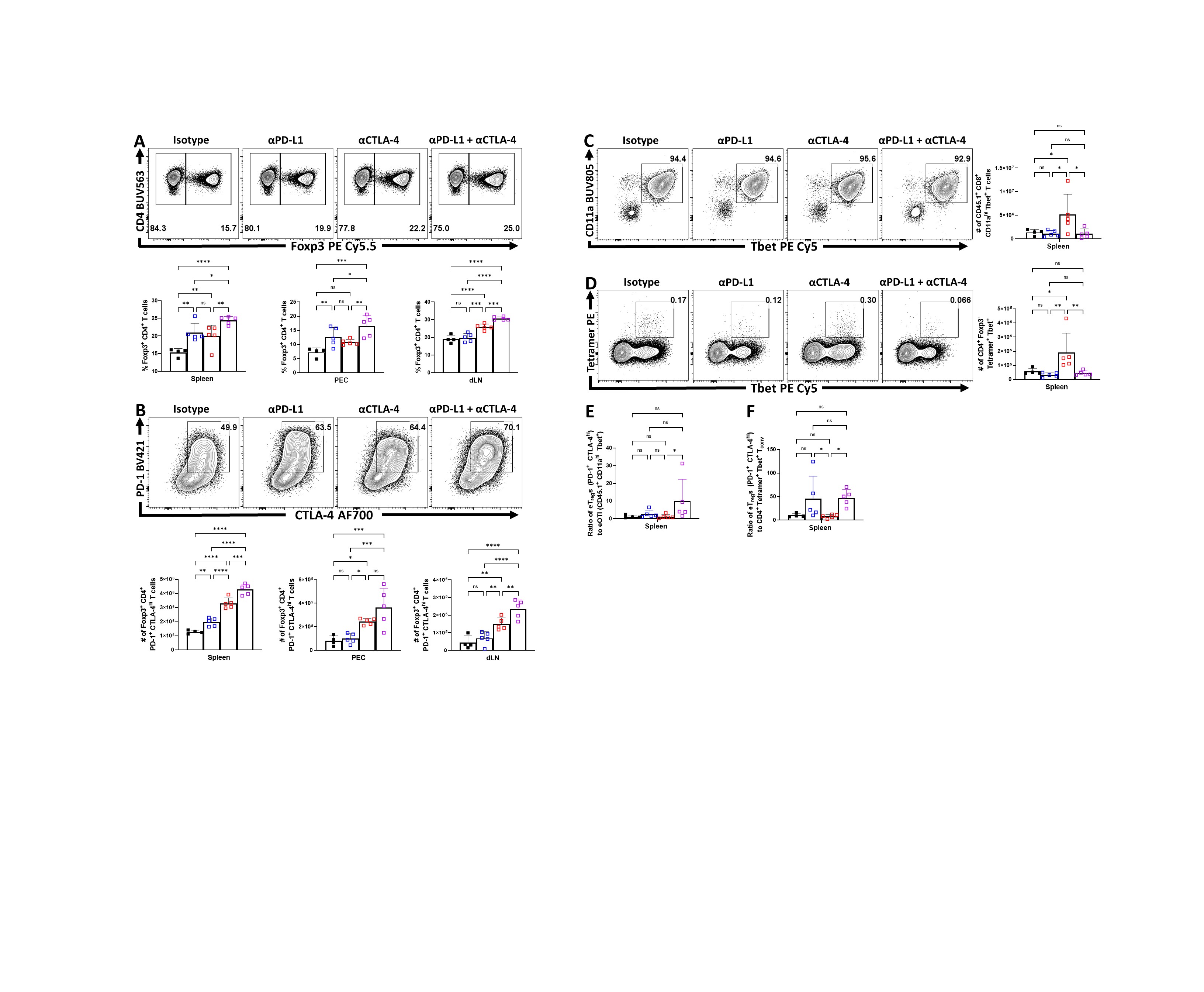

Supplement: Supplementary Figure 5 — Combination blockade does not enhance the formation of T cell immune responses to vaccination, instead results in an increase in the ratio of eTregs: Teff cells. 8 week-old male C57BL/6 mice were given a single intraperitoneal injection of either αPD-L1, or αCTLA-4, or combination αPD-L1 and αCTLA-4, or Isotype control antibody. At 72 hours following treatment, congenically labeled OTI cells were transferred IP, and the hosts were vaccinated with CPS-OVA 24 hours after OTI transfer. The mice were re-dosed with their respective blocking antibody another 24 hours after vaccination. At day 7 following vaccination, the spleens, peritoneal exudate cells (PEC), and draining lymph nodes (dLN) were harvested, and analyzed via flow cytometry. (A) Representative plots of splenocyte-derived bulk CD4+ T cells depicting the Foxp3+ subset from each blockade treatment group and statistically from each tissue harvested (n = 4-5/group, 1-way ANOVA with Fisher’s LSD individual comparisons test, * = p < 0.05, ** = p < 0.01, *** = p < 0.001, **** = p < 0.0001, 1 experimental replicate). (B) Representative plots of splenocyte-derived bulk Foxp3+ Treg cells comparing the number of PD-1+ CTLA-4hi eTreg cells between blockade treatment groups and across each tissue evaluated (n = 4-5/group, 1-way ANOVA with Fisher’s LSD individual comparisons test, * = p < 0.05, ** = p < 0.01, *** = p < 0.001, **** = p < 0.0001, 1 experimental replicate). (C) Representative plots of splenocyte-derived transferred OTI T cells (CD45.1+ CD8+) sub-gated on Tbet+ CD11ahi populations following vaccination (n = 4-5/group, 1-way ANOVA with Fisher’s LSD individual comparisons test, * = p < 0.05, 1 experimental replicate) (D) Splenocyte-derived endogenous (CD45.2+) parasite-specific CD4+ Tconv effector T cell (Teff) responses (Tetramer+ Tbet+) to vaccination (n = 4-5/group, 1-way ANOVA Fisher’s LSD individual comparisons test, * = p < 0.05, ** = p < 0.01, ** = p < 0.01, 1 experimental replicate). (E, F) Graphed rati [file Image_5.jpg]

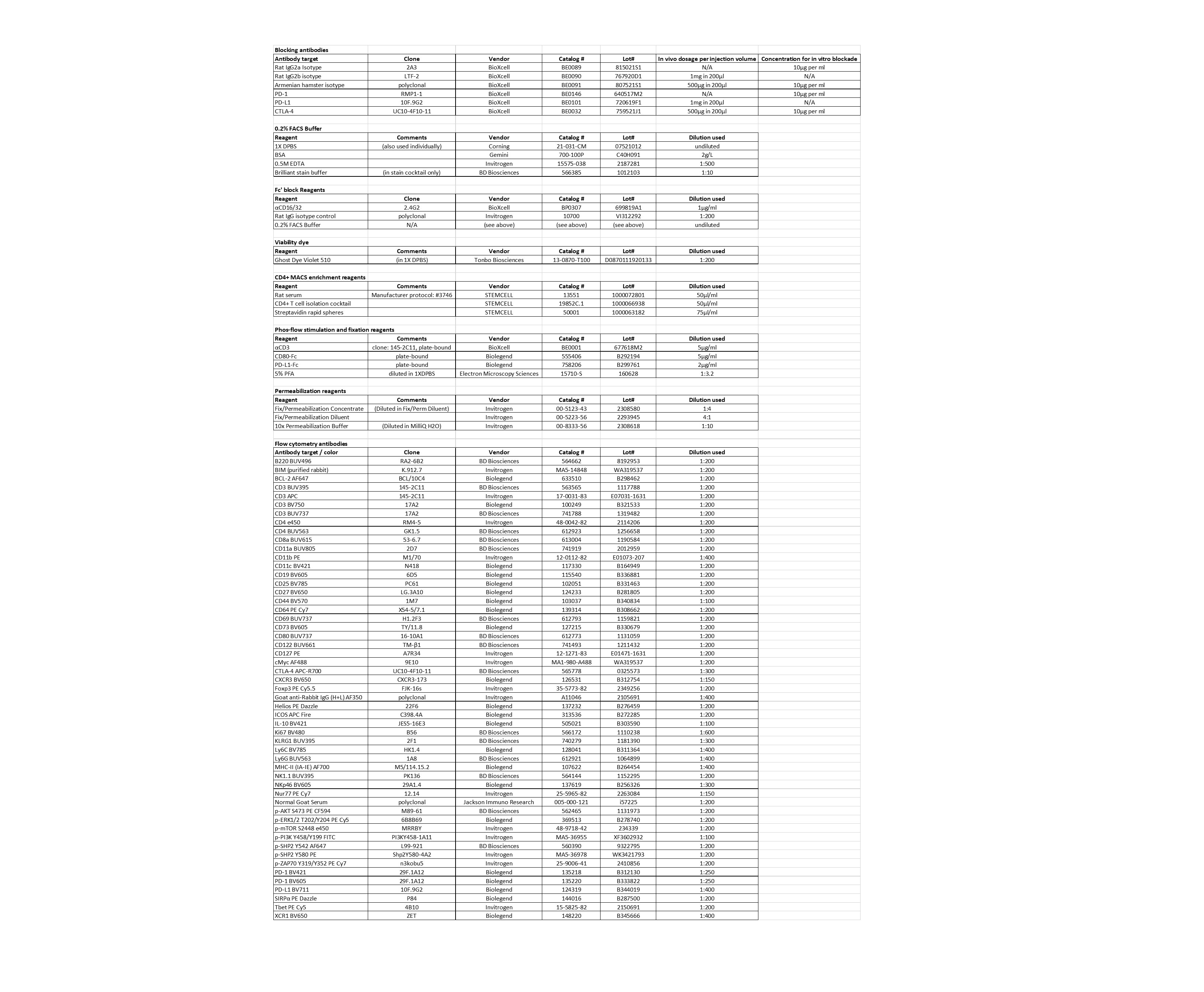

Supplement: Supplementary file 6 [file Image_6.jpg]
